# Methanogenesis from tetramethylammonium and choline in *Methanococcoides methylutens* Q3c requires a nonpyrrolysine monomethylamine methyltransferase homolog

**DOI:** 10.3389/fmicb.2026.1739651

**Published:** 2026-03-19

**Authors:** Jyoti Kashyap, Debdeep Das, Tomislav Ticak, Adam J. Creighbaum, Rakhsha Khatri, Donald J. Ferguson

**Affiliations:** 1 Department of Microbiology, Miami University, Oxford, OH, United States; 2 Department of Biological Sciences, Miami University Regionals, Hamilton, OH, United States

**Keywords:** choline, methylotrophic methanogenesis, methyltransferase, pyrrolysine, tetramethylammonium

## Abstract

Methanogenesis is a key biological process contributing to both global climate change and renewable energy via methane. Methylotrophic methanogenesis is an important, but often underappreciated, component of this process. Recently, there has been an increasing interest in methylotrophic methanogenesis from quaternary amines such as glycine betaine, choline, and tetramethylammonium (QMA). QMA was the original quaternary amine identified as a direct substrate for methanogens and the components of its catabolic pathway were isolated from *Methanococcoides methylutens* NaT1. Loss of strain NaT1 and the absence of genomic sequencing leaves into question the identity of the genes responsible. We previously isolated a related strain of *Methanococcoides methylutens* called strain Q3c, which is capable of growth on the quaternary amines QMA and choline. We conducted proteomic analysis of strain Q3c in which we identified potential targets for QMA and choline metabolism, involving the methyltransferase, MtqB, and its cognate corrinoid protein, MtqC, because they were highly produced in QMA-grown and choline-grown cells differentially compared to trimethylamine grown cells and the 82.8 and 96.4%, identity of their N-termini, respectively, to MtqBC identified previously in NaT1. Surprisingly, MtqB was identified as a nonpyrrolysine monomethylamine methyltransferase (MtmB) homolog. This was validated via enzymatic assay and molecular co-docking of MtqB with QMA and choline along with its cognate partner MtqC, representing the first ever function for a non-Pyl MtmB demonstrated. The phylogeny of MtqB and MtqC showed unique clustering of these proteins compared to other homologs, suggesting these proteins may be important in our continued study of the evolution of Pyl- and non-Pyl methyltransferase systems.

## Introduction

The biological production of methane by archaea, or methanogenesis, is responsible for generating significant quantities of methane that play an important role in biogeochemical cycles and climate change (IPCC AR6). Methane is second only to carbon dioxide as a greenhouse gas in terms of its impact on climate change (Lyu et al., 2018; Kountz et al., 2020). Methane is estimated to be responsible for 20% of the increase in global warming and methanogenesis contributes up to 70% of the total methane produced (Lyu et al., 2018). Some major sources of atmospheric biological methane emissions are related to livestock, sewage treatment plants, wetlands, landfills, and the ocean (Leng et al., 2018). Methanogenesis is a double-edged sword where it plays a critical role in the earth’s biogeochemical cycles and can produce a renewable energy source but also contributes to climate change. Methanogens are responsible for generating 500 million tons of methane gas annually, thereby contributing significantly to global warming and climate change (Pandey et al., 2015) and we have yet to have a full appreciation of their metabolic range.

**Figure 1:**
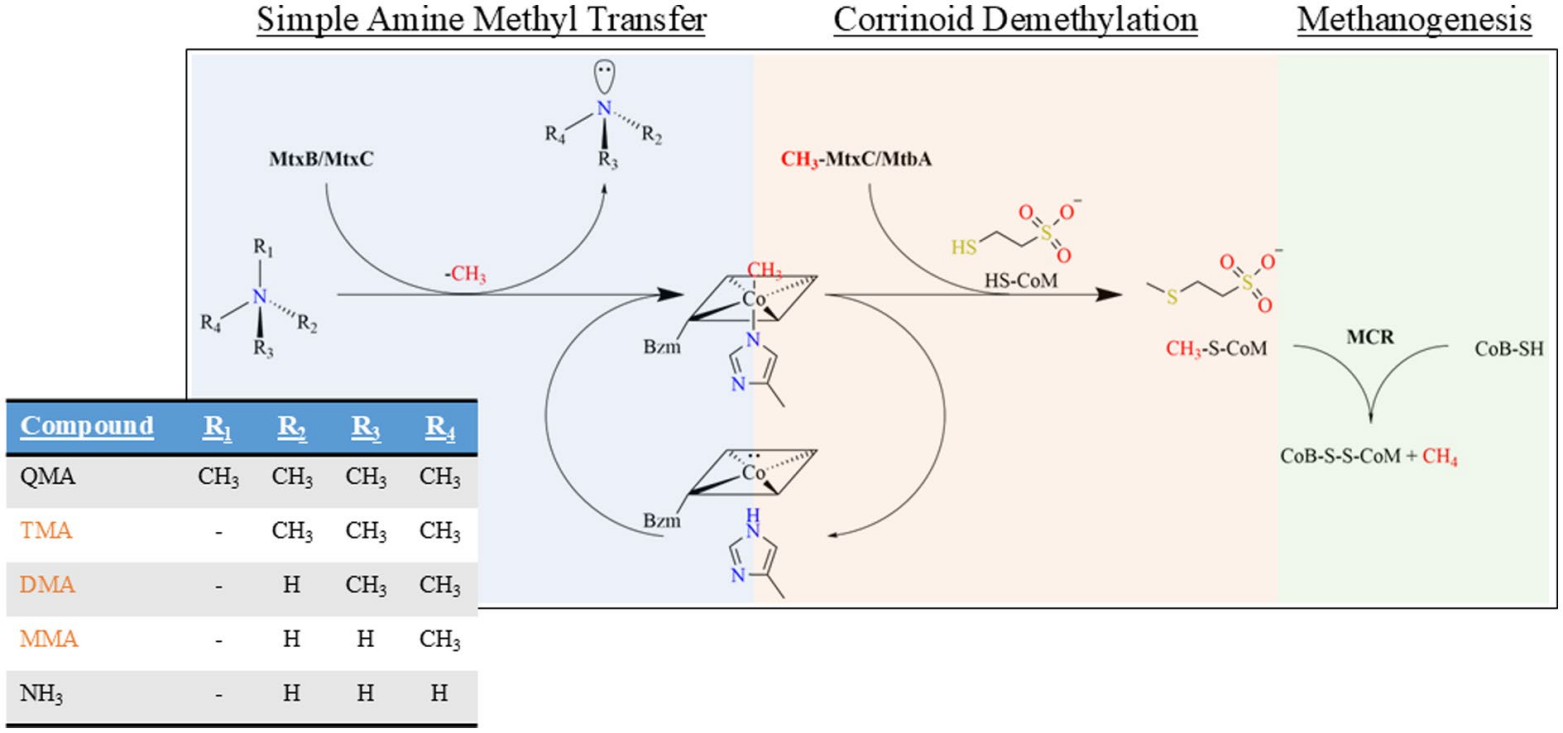
Q3c methanogenic methylamine pathway schema of non-Pyl and Pyl-containing MtxBs from QMA. Methanogenesis initiation via QMA demethylation through MtqB interaction with MtqC in the Co(I) oxidation state to generate both TMA and form methylated MtqC in the Co(III) state (blue section). Subsequently, methylated MtqC acts as the substrate for MtbA to generate methyl-CoM (orange section). The methyl-CoM serves as a substrate along with coenzyme B for methyl coenzyme methylreductase (MCR) to form the heterodisulfide CoB-S-S-CoM molecule and methane (green section). Further demethylation of TMA, DMA, and MMA to ammonia then proceeds via Pyl-containing MtxBs and cognate MtxCs (Supplementary Figure 1).

In marine sediments, methylamines serve as important substrates involved in environmental methane production. While various microorganisms can utilize methylated compounds like monomethylamine (MMA), dimethylamine (DMA), and trimethylamine (TMA), aerobically through different mechanisms, in anoxic marine environments these compounds are often preferentially metabolized by methanogens through encoded methyltransferases (Figure 1) as they face limited competition from sulfate-reducing bacteria, which primarily compete for hydrogen and acetate (Oremland and Polcin, 1982; King, 1984). Several methanogenic lineages of *Methanosphaera* and *Methanomassiliicoccus* have the capability to perform hydrogen-dependent methylotrophic methanogenesis, additionally, lineages of *Methanococcoides* and *Methanolobus* are involved in utilization of quaternary amines (QAs) (Watkins et al., 2012; Ticak et al., 2015).

These direct QA demethylation pathways, while unique in their substrate specificity, ultimately feed into the core methylotrophic methanogenesis pathways. The general methylotrophic methanogenesis pathway is comprised of a 3-component system: (1) initiation by the first substrate-specific methyltransferase that transfers the methyl group from the substrate onto the second component, (2) a cognate corrinoid binding protein (CBP) in the highly reduced cobalt state Co(I), and finally (3) the secondary methyltransferase leading to the penultimate step of transferring the methyl group from the methylated CBP onto coenzyme M (CoM) that will finally lead to CH_3_ reduction to CH_4_ (Figure 1). Additionally, two other staples are essential for methylamine methanogenesis: (1) a reductive activation enzyme, RamA (Huening et al., 2021), that catalyzes the ATP-dependent reduction of the CBP from the inactive Co(II) state to the active Co(I) state and (2) utilization of the 22nd canonically encoded amino acid *L*-Pyrrolysine (Pyl) in the substrate-specific methyltransferase. The importance of Pyl for methylamine methyltransferase functionalization has been demonstrated (Hao et al., 2002) and its biosynthetic genes are typically near methylamine associated genes where it likely plays a role in control of methylamine metabolism, but recent studies show that Pyl is absent and not required in methyltransferases involved in methanogenic QA metabolism (Creighbaum et al., 2019).

Understanding of QA metabolism in anaerobic environments has advanced significantly in the past decade. While QAs were historically thought to be primarily fermented by bacteria to TMA before entering methanogenic pathways (King, 1984), recent research has revealed direct QA demethylation pathways in both methanogens and bacteria, leading to no production of TMA (Kountz et al., 2020; Ticak et al., 2014; Creighbaum et al., 2019; Ellenbogen et al., 2021; Jiang et al., 2025; Picking et al., 2019). This paradigm shift began with the discovery of a non-Pyl glycine betaine (GB) methyltransferase in *Desulfitobacterium hafniense* Y51 (Ticak et al., 2014), followed by identification of similar direct QA demethylation systems in *Methanolobus vulcani* B1d (Creighbaum et al., 2019) for methanogenesis from GB, which belong to the Cluster of Orthologous Genes 5,598 (COG5598). The similarity and diversity of QA-utilizing pathways is further exemplified by recent characterizations of novel anaerobic methyltransferases that target various QA substrates, including *L*-carnitine (Kountz et al., 2020), *γ*-butyrobetaine (Ellenbogen et al., 2021), proline betaine (Picking et al., 2019), and phosphocholine (Jiang et al., 2025). Our expanded understanding of the substrate range, ecological significance, and evolution begs the question: are these COG5598 methyltransferases responsible for all QA-dependent methanogenic pathways?

*Methanococcoides methylutens* Q3c (Q3c) is an estuarine isolate that can degrade the QAs tetramethylammonium (QMA) and choline. Strain Q3c is one of the few isolated methanogens known to degrade these QAs (Ticak et al., 2015). QAs have long been known to be broken down into TMA, which contributes significantly to methylotrophic methanogenesis (King, 1984), however, this is unlikely for direct choline demethylation but in the context of QMA, TMA is the next downstream intermediate. The first quaternary amine-dependent methanogenesis pathway reported was for QMA:CoM isolated from *Methanococcoides methylutens* NaT1 (previously known as *Methanosarcina* sp. NaT1); this strain has since been lost but it is the only known methanogen from which the QMA demethylation activity has been biochemically demonstrated via pure isolated proteins (Asakawa et al., 1998; Kazuhiro Tanaka, 1994). Despite the discovery of the enzymes involved in QMA demethylation in NaT1, the loss of the strain leaves a gap in understanding the genes responsible except for leaving behind only minimal information in the form of N-terminal sequences of MtqB, MtqC, and MtqA.

Given the void left by the loss of NaT1 as well as the recent advances in our understanding of QA metabolism, we now have an opportunity to gain more appreciation of QA utilization in the environment and an opportunity for discovering the enzymes responsible for QMA and choline dependent methanogenesis. All currently identified corrinoid-dependent QA methyltransferases belong to the COG5598 family of TMA methyltransferases (MttBs). Based on these findings and the presence of three non-Pyl MttBs in the Q3c genome belonging to the COG5598 superfamily, we initially hypothesized that Q3c would utilize non-Pyl MttBs for choline and QMA demethylation. However, our assessment of the N-terminal sequences and subsequent analyses presented in this study provide strong evidence that the QMA and choline methyltransferase is a singular non-Pyl monomethylamine methyltransferase (MtmB) homolog rather than differential non-Pyl MttBs. This unexpected discovery suggests the first physiological context of a non-Pyl MtmB in either archaea or bacteria that may contribute significantly to environmental QA degradation and methane biogenesis. QMA was initially evaluated due to contamination from industrial wastewater, however, the finding of this same pathway divergent from Tokyo Bay suggests other more widespread environmental and evolutionary context for QMA and discovery of wide-spread choline demethylation that has been underappreciated to date.

## Methods

### Culturing of *Methanococcoides methylutens* Q3c

Culturing was performed in brackish medium, as described previously (Ticak et al., 2015). The following components were added in an anaerobic chamber after exchanging the headspace with CO_2_/N_2_ (20/80%): 1 M KH_2_PO_4_, NH_4_Cl, Cysteine-HCl, 3% Na_2_S-9H_2_O. After the medium was reduced, it was aliquoted (10 mL) to 27 mL Balch tubes and sealed with butyl stoppers and seal crimped. The headspace of the tubes was exchanged with CO_2_/N_2_ (20/80%) gas mix on a vacuum and gas exchange manifold system with three 1 min cycles, prior to autoclaving. The culturing conditions per 10 mL Balch tubes were described previously (Ticak et al., 2015) with 30 mM QMA, TMA, or choline, and inoculum (0.1%). Growth was measured at 600 nm using a direct insert adapter for Balch tubes for Spectronic 20 (Thomas Scientific). Cultures were adapted to each carbon source (choline, QMA, and TMA) through at least three sequential transfers. Each cell transfer was grown to mid-log phase prior to inoculating fresh medium with 0.1% (v/v) culture. Following adaptation to desired culture conditions, cultures were maintained by continuous sub culturing under the same conditions mentioned above and stored as glycerol stocks (10% final glycerol) at −80° C.

### Genome isolation, sequencing, and annotation of *Methanococcoides methylutens* Q3c

The genomic DNA of Q3c was isolated initially via phenol-chloroform extraction and evaluated for high quality, non-degraded, molecular DNA through 0.5% TAE gel electrophoresis. Extracted DNA was then submitted initially for MiSeq (Miami University) and then followed up with analysis through PacBio (University of Delaware) sequencing yielding one contig. Rapid Annotation using Subsystem Technology (RAST)^1^ (Aziz et al., 2008) was used as the initial annotation platform following genomic sequencing, prior to submission to National Center for Biotechnology Information (NCBI) (Accession #: NZ_CP199767.1). During the submission, annotation was added by the NCBI Prokaryotic Genome Annotation Pipeline (PGAP). The strain was deposited in the Biological Resource Center, National Institute of Technology and Evaluation (NBRC) Culture Collection for preservation and further study and cultivation.^2^

### Proteomics

Mid-log phase cultures of Q3c were grown on QMA (30 mM), choline (30 mM), or TMA (30 mM) in triplicate. The cultures were grown anoxically in 1 L flasks and centrifuged at 15,344 x *g* at 4 °C for 15 min to collect pellets, which were resuspended in 10 mL of lysis buffer containing 50 mM Tris–HCl at pH 8.0, beta-DDM detergent and Halt protease inhibitor cocktail (Thermo Fisher Scientific). The resuspended cells were lysed three times at 20,000 psi using a French press. Protein concentrations were measured using a Bradford assay kit (Thermo Fisher Scientific). For each sample, 100 μg of protein was used followed by the addition of 8 M urea and 10 mM DTT. Iodoacetamide and Tris–HCl were added before the gold trypsin (Promega) digestion at 37 °C for 19 h. The protein cleanup was done using C18 Sep-Pak columns (Waters Corporation). The proteins were fractionated using the Thermo Fisher Scientific fractionation kit (Catalog number 84868) into eight fractions with varying concentrations of acetonitrile and 0.1% triethylamine. These fractions were separated using nano high performance liquid chromatography (EASY-nano LC 1000 from Thermo Fisher Scientific) coupled with LTQ Orbitrap XL Mass Spectrometer (Thermo Fisher Scientific). The peptide range was 350–1800 m/z, resolution of 30,000 in data-dependent mode. The 12 most abundant peaks were subjected to MS/MS collision induced dissociation fragmentation. The proteins obtained were matched against the Q3c proteome using Pattern Lab (Carvalho et al., 2016). Proteins were identified by matching MS/MS spectra against the *M. methylutens* Q3c proteome database. Relative protein abundances were quantified using normalized spectral abundance factors (NSAF) to account for protein length and total spectral counts. Differential protein abundance between growth conditions was assessed using the PatternLab t-Fold framework, which integrates fold-change analysis with statistical testing and applies false discovery rate (FDR) correction to generate q-values. Differential abundance was evaluated across three independent biological replicates. The data have been included in the Supplementary Material and deposited in the MassIVE database at the following address.^3^

### Phylogenetic reconstruction of the MtmB/MtxC superfamily

The Pyl-encoding MtmB1 from *Methanosarcina barkeri* (NCBI – AAC38636) was used as a query to acquire other Pyl-encoding MtmB sequences via tBLASTn (McGinnis and Madden, 2004). Then the amino acid sequence of MtqB (ACT9XH_RS04775) from *Methanococcoides methylutens* Q3c was used in PSI-BLAST (Altschul and Altschul, 1997) to obtain other non-Pyl MtmB sequences. We eliminated sequences below a 25% identity or those that were greater than one standard deviation from the mean amino acid length relative to MtqB. The 25% cutoff was chosen to obtain a wide breadth of enzymes due to the lack of knowledge regarding non-Pyl MtmB homologs and their function. Sequences were further processed by performing trimming of total sequences with CD-HIT (Li and Godzik, 2006) to remove all sequences with greater than 90% identity. The final file consists of 1,061 sequences with a 459 average amino acid length. Sequences were aligned with MUSCLE version 5.0 (Edgar, 2022) with default parameters. FastTree2 (Price et al., 2010) was used to approximate maximum likelihood across the MtmB family using JTT + CAT (Price et al., 2010) with gamma distribution. Phylogeny was analyzed and edited with the interactive Tree of Life (iTOL) (Letunic and Bork, 2021). The same methodology was used for MtxC proteins, using ACT9XH_RS04770 (MtqC), which netted a final file of 2,952 corrinoid binding proteins.

### Molecular modeling and ligand docking of ACT9XH_RS04770, ACT9XH_RS04775, and ACT9XH_RS07150

I-TASSER (Roy et al., 2010; Yang et al., 2014; Zhang, 2008) and MODELLER (Krieger, 2012; Krieger et al., 2009) were used to thread the protein sequence of ACT9XH_RS04775 (MtqB), and MtqB clade members then directly compared to both crystal structure of the monomethylamine methyltransferase in PDB with accession 1NTH (Hao et al., 2002; Hao et al., 2003) and 1L2Q (Hao et al., 2002; Hao et al., 2003). The MtqB clade members along with MtmB were aligned and inspected for residues within 4–5 Å from QMA and choline to identify proposed active site signatures, generated with WebLogo3 (Crooks et al., 2004). Molecular docking predictions with ligands were performed based on probable relevance to Pyl and Pyl-ammonium adduct positioning in the MtmB homologs using AutoDock Tools (Ramsbottom et al., 2018), AutoDock Vina 1.2 (Eberhardt et al., 2021) for both targeted and unbiased docking along with Boltz-2 (Passaro, 2025). The specifications for targeted docking was a value of center_x, center_y, and center_z of 63, 65, and 67, respectively, and an associated size of 20 with AutoDock Vina. Multiple poses (Huening et al., 2021; Hao et al., 2002; Ticak et al., 2014; Creighbaum et al., 2019; Ellenbogen et al., 2021; Jiang et al., 2025) were generated for both Vina and Boltz-2 in an unbiased manner, and the best fit was chosen for representation. Similar methods were used for ACT9XH_RS04770 (MtqC) and ACT9XH_RS07150 (MtqA) structures. RMSD measurements were performed with MtqB bound to either choline or QMA compared to MtmB (1L2Q). Two pairs of co-dockings were performed; (1) ACT9XH_RS04770 (MtqC)/ACT9XH_RS04775 (MtqB), and (2) ACT9XH_RS04770 (MtqC)/ACT9XH_RS07150 (MtqA) each with their respective ligands using Boltz-2 and visualized with PyMol v2.3 (The PyMOL Molecular Graphics System, Version 2.3 Schrödinger, LLC.).

### Cloning and expression of the putative *mtqB* in *Escherichia coli*

The gene encoding ACT9XH_RS04775 (MtqB) was codon optimized, synthesized, and cloned into the pET28b vector by GenScript^4^ to generate the pET28_*mtqB* plasmid. *E. coli* DH5-*α* and BL21(DE3) cells were then chemically transformed with the pET28_*mtqB* plasmid for plasmid maintenance and expression, respectively. For gene expression, a freshly grown colony of transformed BL21(DE3) cells was picked and grown overnight at 37 °C while shaking in 5 mL of LB supplemented with 50 ug/ml kanamycin and then transferred to 1 L of LB-Kan and grown at 37 °C while shaking until it reached an OD_600_ of 0.4–0.6 and then induced with 0.08 mM isopropyl *β*-D-1-thiogalactopyranoside (IPTG) and incubated at 14 °C overnight. Cells were then harvested and lysed as described above after resuspension in buffer A (50 mM sodium phosphate, 300 mM NaCl, 40 mM imidazole, pH 7.4) at a concentration of 3 mL per g cell.

### Partial purification of ACT9XH_RS04775

Extracts of *E. coli* BL21(DE3) prepared as described above were loaded onto a 2 mL Ni-NTA (Thermo Fisher Scientific) gravity flow column. The column was then washed with 5 mL of buffer A and eluted with 2 mL of buffer B (50 mM sodium phosphate, 300 mM NaCl, 500 mM imidazole, pH 7.4).

### Free cob(I)alamin and coenzyme M methylation assays

Assays to examine substrate dependent coenzyme M methylation in cell-free extracts of *M. methylutens* Q3c were performed as described previously (Creighbaum et al., 2019). Assays to measure substrate dependent methylation of free cob(I)alamin spectrophotometrically were performed as described previously (Creighbaum et al., 2019) except that the assay was performed at room temperature in a 1 cm pathlength cuvette. The assay was initiated by the addition of 200 μL (approximately 50 μg) of partially purified enzyme. The assays were performed using choline, QMA, MMA, TMA, or glycine betaine as methyl donor substrates as well as no substrate controls.

## Results

### Genome analysis

**Figure 2:**
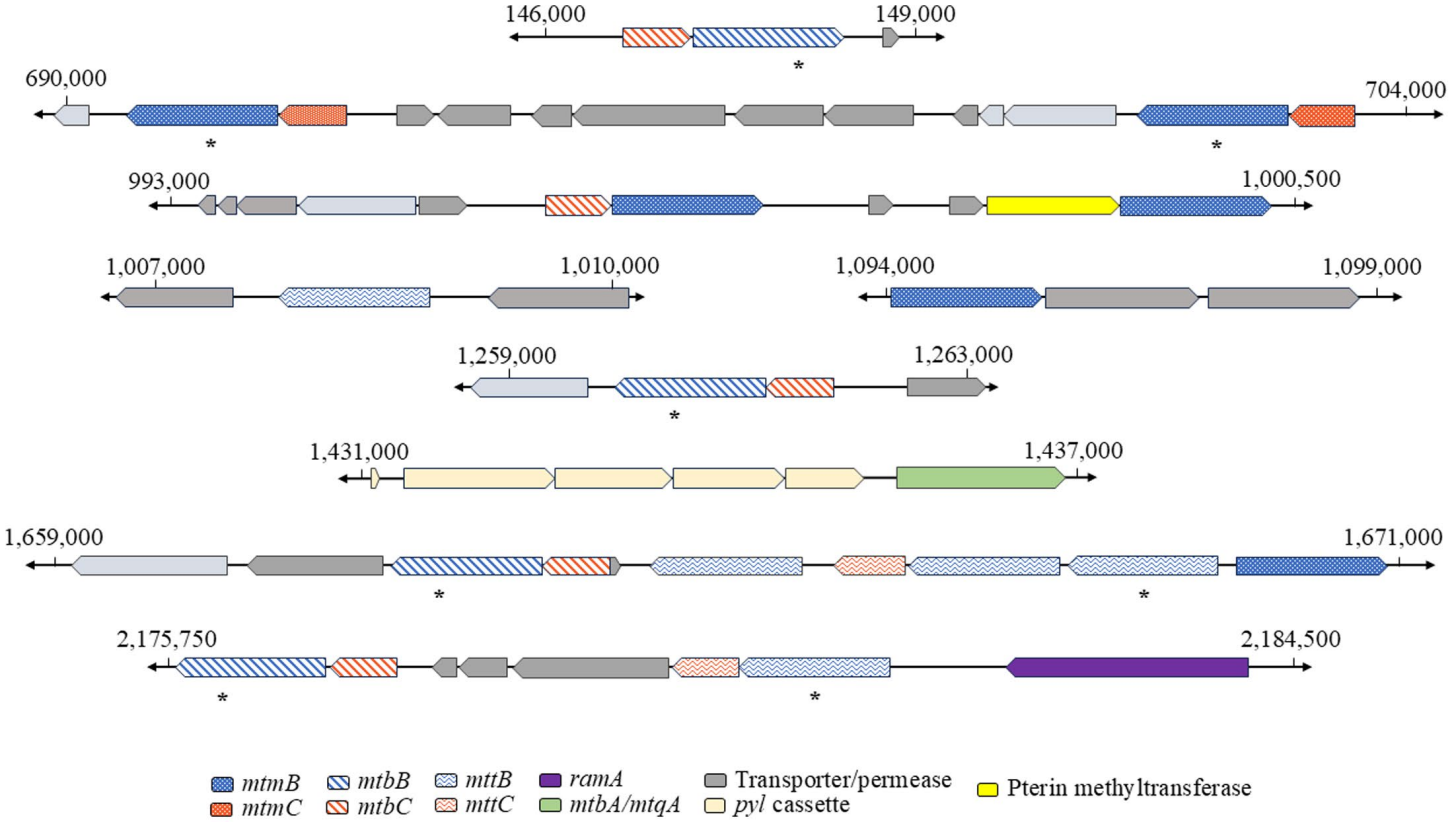
Genomic context of *Methanococcoides methylutens Q3c* methylamine methyltransferases pathways. Genes encoding members of methylamine methyltransferases, *mtxB* (blue), and cognate corrinoid proteins, *mtxC* (orange), families are represented above with relevant upstream and downstream genes across the Q3c genome. Those genes are classified by specific substrate annotations as monomethylamine (dots), dimethylamine (diagonal stripes), and trimethylamine (waves) genes. Pyrrolysine, across the *mtxBs* where a TAG stop codon is positioned, are represented approximately via asterisk where appropriate.

The genome of Q3c was sequenced at the University of Delaware DNA Sequencing and Genotyping Center, using the PacBio platform and analyzed with prior genomic scaffolding from MiSeq. Q3c has a closed genome size of 2,584,604 bp with 320× coverage.^5^ We observed the presence of three non-Pyl *mttB* genes in the genome (Figure 2), that we speculated were responsible for QMA demethylation in Q3c. Interestingly, there was the observation of several non-Pyl *mtmB* genes, with one unique member being downstream of a predicted dimethylamine corrinoid-binding protein (*mtbC*) gene, a finding that is uncommon among observed genomes from methylamine amine utilizing organisms (Gaston et al., 2011; Ferguson Jr et al., 2000). The sheer number of divergent *mtxB*s suggests there are additional substrates that Q3c can utilize, which are yet to be discovered.

### Exploration of the enzymes of QMA- and choline-CoM methyl transfer pathway

**Figure 3:**
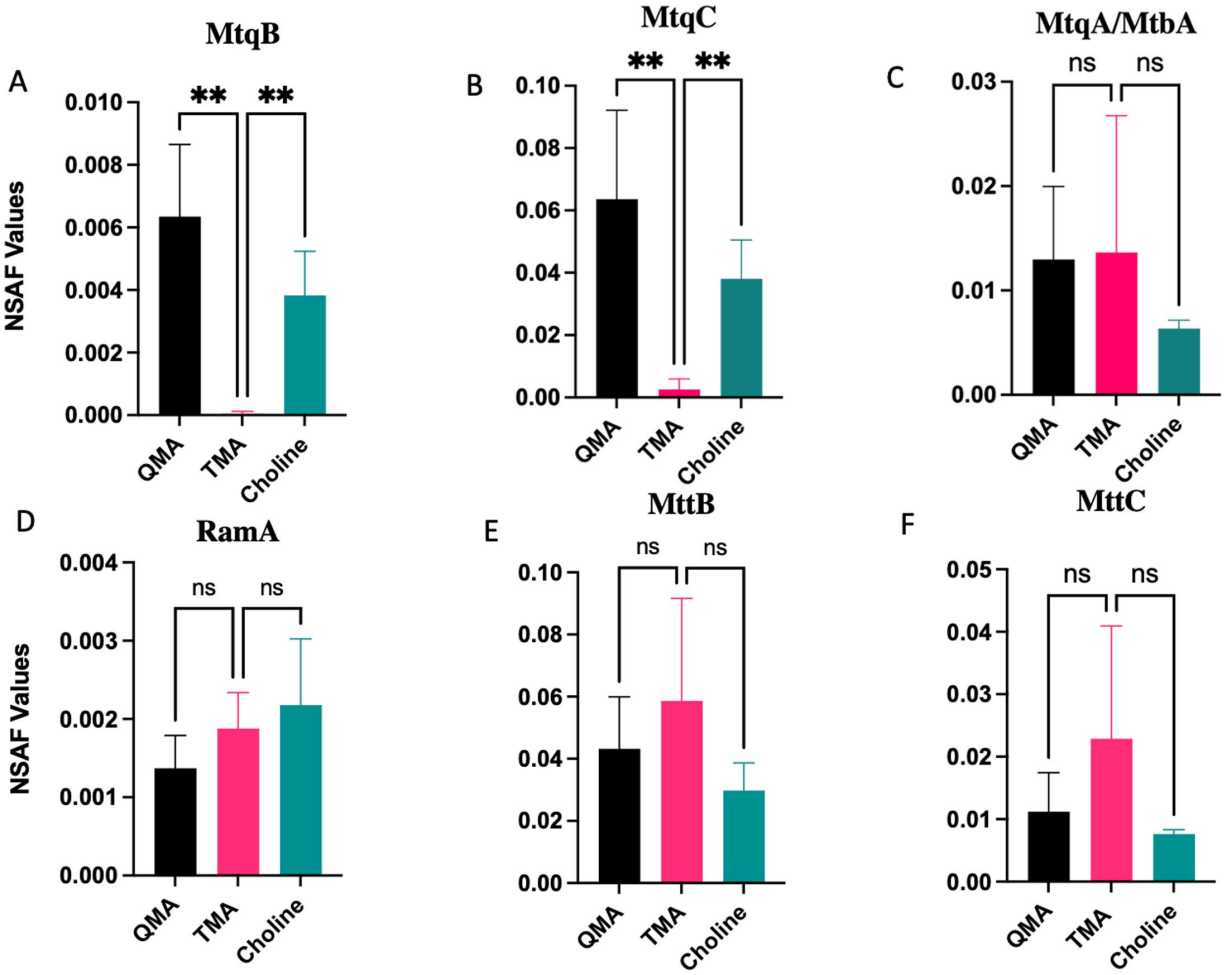
Differential abundance of methylamine-utilization proteins in Q3c cells grown on QMA, choline, or TMA. Normalized spectral abundance factor (NSAF) values are shown for key proteins involved in methylated amine utilization: **(A)** MtqB (ACT9XH_RS04775; QMA methyltransferase), **(B)** MtqC (ACT9XH_RS04770; QMA corrinoid-binding protein), **(C)** MtqA/MtbA (ACT9XH_RS07150; methyltransferase), **(D)** RamA (ACT9XH_RS10715; corrinoid reductive activation protein), **(E)** MttB (ACT9XH_RS10710; TMA methyltransferase), and **(F)** MttC (ACT9XH_RS10705; TMA corrinoid-binding protein). Bars represent mean NSAF values from three independent biological replicates (*n* = 3), with error bars indicating SD. Statistical significance was determined using ordinary one-way ANOVA followed by Dunnett’s multiple comparisons test (pairwise comparisons indicated by brackets). Significance is denoted as ns (not significant), **p ≤ 0.002, ***p ≤ 0.0002, ****p ≤ 0.0001.

**Figure 4:**
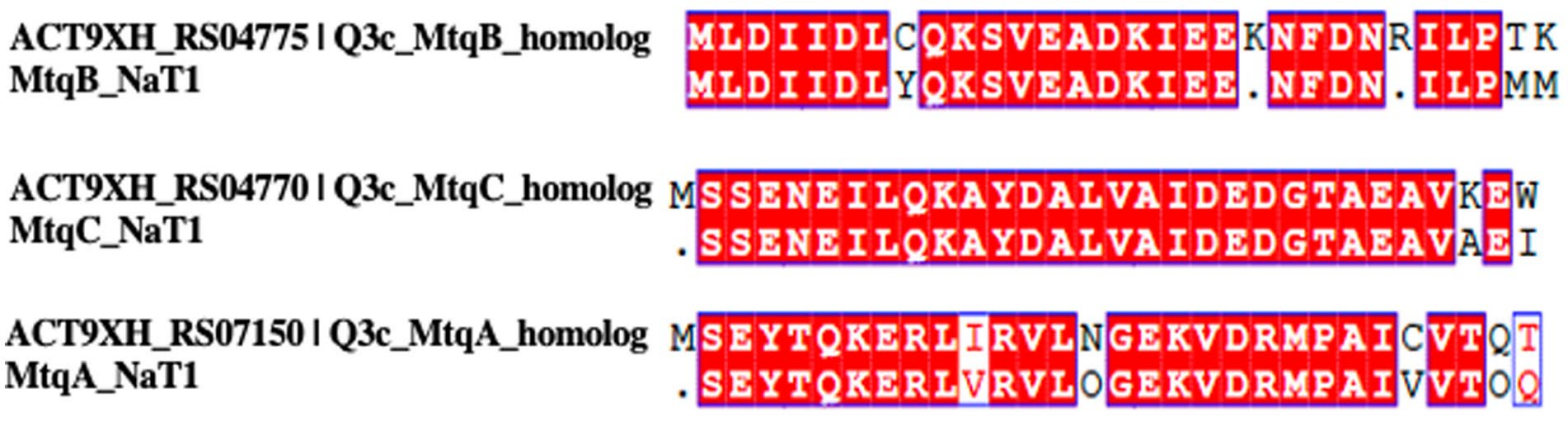
N-terminal sequence alignments of methyltransferases and corrinoid binding protein from Methanococcoides methylutens strains NaT1 and Q3c. The QMA methyltransferase MtqB from strain NaT1 shows 88.9% identity with ACT9XH_RS04775 from Q3c. The corrinoid protein MtqC exhibits 96.4% identity with ACT9XH_RS04770, the highest conservation among the three components. The methylcorrinoid:coenzyme M methyltransferase MtqA displays 82.8% identity with ACT9XH_RS07150. Red boxes with white letters indicate identical residues, and residue numbers are shown above each alignment. Blue boxes outline regions of similarity. Alignments were generated using Clustal Omega and visualized with ESPript 3.0.

To investigate the proteins responsible for QA demethylation, we performed proteomic analysis with Q3c grown under three different substrates as the sole energy sources (Figure 3, Supplementary Figure 1). When Q3c was grown in the presence of QMA as the sole energy source, we predicted that the non-Pyl MttBs would be among the most abundant proteins in the proteomic data. To our surprise, we saw the occurrence of the non-Pyl MtmB homolog ACT9XH_RS04775 being among the most abundant methyltransferases produced during growth on QMA and choline, but not on TMA (Figure 3). As non-Pyl MtmB functions have yet to be elucidated, it was peculiar that ACT9XH_RS04775 was the only non-Pyl methyltransferase produced during growth on these carbon sources. Upon analyzing the sequence of ACT9XH_RS04775, we observed a high degree of identity of 82.8% with the reported N-terminus of the QMA methyltransferase (MtqB) from *Methanococcoides methylutens* NaT1 (Figure 4) (Asakawa et al., 1998) and the full enzyme shares 87 and 78% identity to non-Pyl MtmB homologs from *Methanococcoides* strains SA1 and AM1, of which AM1 is reported to be a QMA/choline utilizing methanogen. For the sake of comparison, we also aligned the MtqB N-terminus from NaT1 to a Pyl-MtmB sequence from Q3c which showed a poor alignment with only 25% identity (data not shown). Additionally, the N-terminus of the corrinoid protein (MtqC) showed a high degree of identity of 96.4% with ACT9XH_RS04770, a corrinoid binding protein from Q3c, which was likewise abundant in QMA- and choline-grown cells but not in TMA-grown cells (Figures 3, 4). The second methyltransferase MtqA in strain NaT1 also showed a high degree of identity of 88.9% with the N-terminal sequence of ACT9XH_RS07150, an amine-specific methylcorrinoid:coenzyme M methyltransferase (MtbA) in Q3c (Figures 3, 4). The strain NaT1 has been lost and no QMA methyltransferases have since been characterized to date and although phosphocholine activity has been reported (Jiang et al., 2025) it does not involve a non-Pyl MtmB.

### Docking and phylogeny of ACT9XH_RS04775

**Figure 5:**
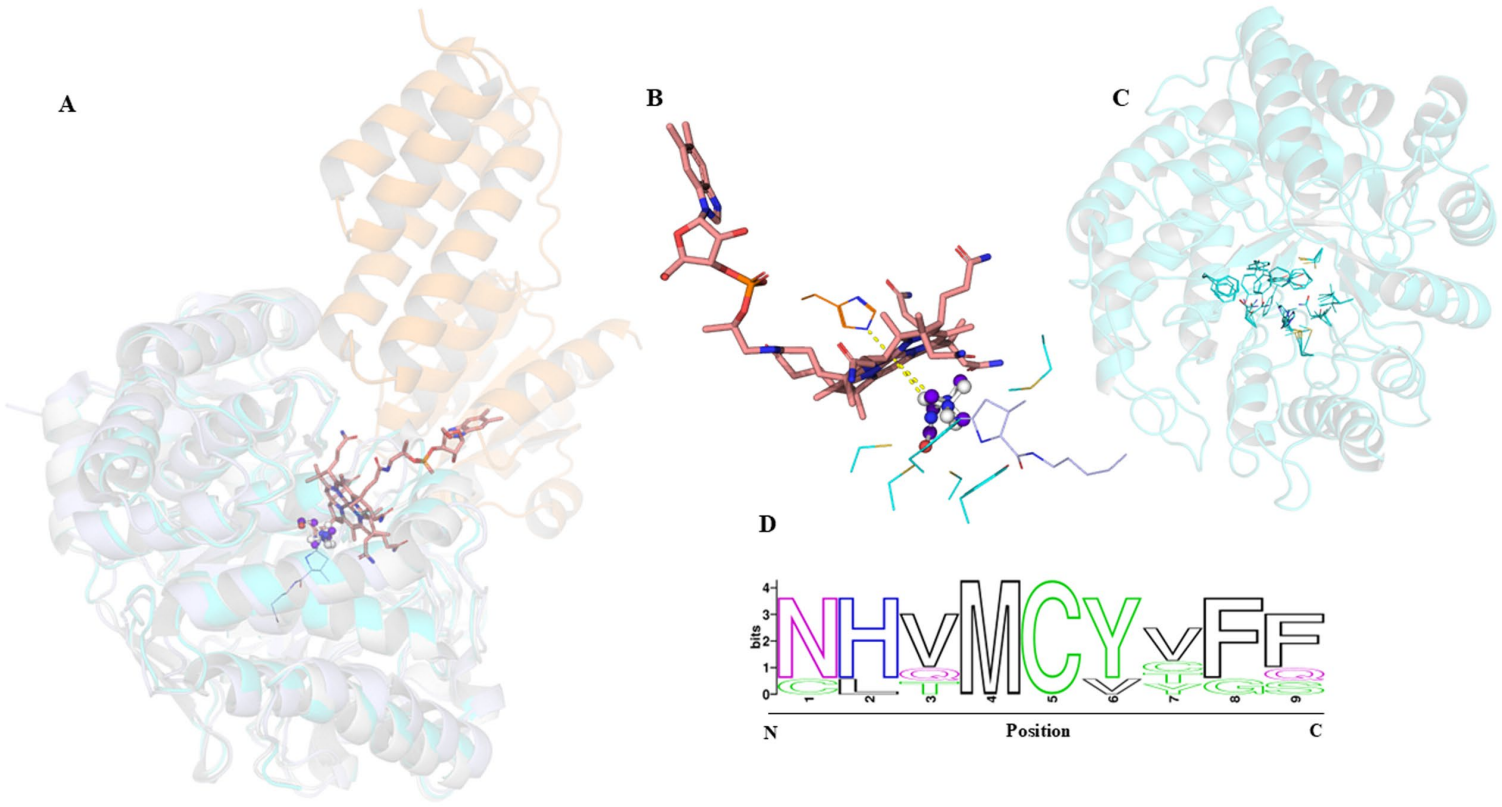
Docked structures of the proposed QMA/choline methyltransferase corrinoid co-structure overlays near Pyl-ammonium adduct and liberated NH_3_ MtmB structures. **(A)** An *in silico* structure of the tetramethylammonium methyltransferase, ACT9XH_RS04775, (cyan) docked simultaneously to both tetramethylammonium (white ball and stick) and cobalamin (pink) bound ACT9XH_RS04770 (orange) aligned to the MtmB crystal structures, 1NTH and 1L2Q (slate). This was also performed with choline (purple blue). The total alignment of the MtmB/MtqBC complex is given as RMSD of 1.201 across the protein topology while the RMSD between MtmB and MtqB is 0.149. **(B)** Close up analysis of the QMA/B_12_ or choline/B_12_ MtqBC, highlighting both a high degree of sulfur containing and aromatic residues ranging from 2.7–4.2 angstroms. The distances of the Pyl-ammonium adduct to QMA stands at 0.8 angstrom while ammonium (blue sphere) is 1.2 angstrom. The proposed leaving methyl group of QMA and Cob(I)alamin distance measures at 3.2 angstroms while choline and cob(I)alamin is 3.5. **(C)** Structural models of the lower phylogenetic branch in Figure 6 compared to the frequency of residues (represented in **D**) for conservation and mechanistic understanding toward either corrinoid-binding proteins or tetramethylammonium with likelihood being represented in bits.

To further explore the interaction of ACT9XH_RS04775 with QMA and choline, we performed molecular docking experiments in silico both alone and with its MtqC partner. By examining how QMA and/or choline docks to ACT9XH_RS04775, we can compare the predicted active site to the known crystallographic structure of the MMA methyltransferase, MtmB (Figures 5A,B). The close distances of the nitrogen atom of both choline and QMA to post-MMA demethylation, i.e., Pyl-ammonium adduct, stands at 0.8 angstrom for QMA and 1.0 angstrom for choline. While the release of ammonium after methyl transfer resides at 1.2 angstrom for QMA and 2.1 for choline (Figure 5B). Furthermore, looking at the proposed methyl leaving groups and the cobalamin cofactor, the distance is 3.2 angstrom (QMA) or 3.5 angstrom (choline) which is feasible for activity. The closeness of the 1L2Q crystal compared to the QMA-docked MtqBC complex highlights an interesting observation toward further examples of non-Pyl/Pyl molecular mimicry, originally proposed for GB in MtgB (Ticak et al., 2014; Creighbaum et al., 2019). Initial docking studies for QMA in ACT9XH_RS04775 indicated a kcal/mol of −2.1, a kcal/mol of −2.9 for choline, and a value of −0.2 kcal/mol for MMA, indicating a more thermodynamically favorable interaction with choline vs. QMA and a possibility for very residual MMA activity.

**Figure 6:**
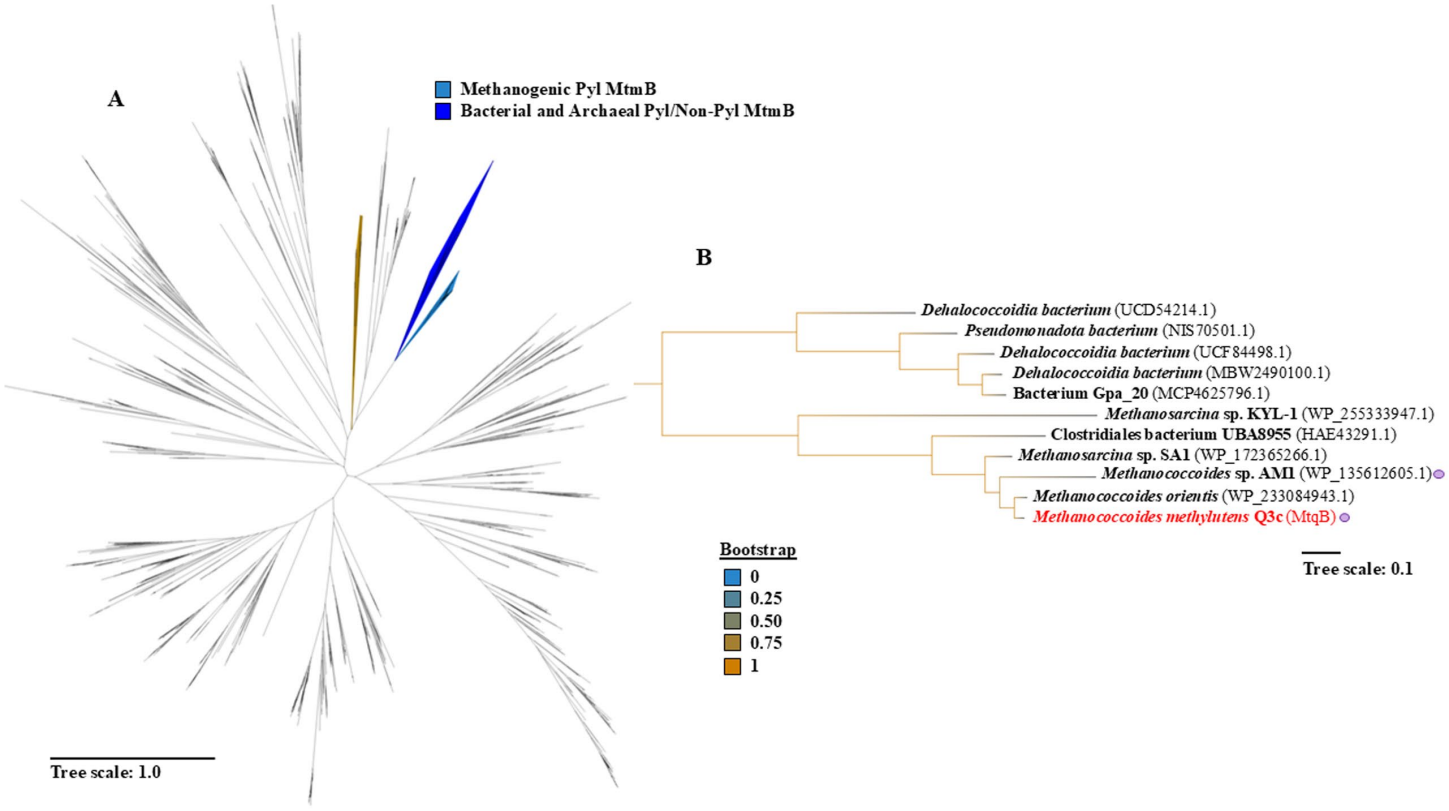
Proposed tetramethylammonium/choline methyltransferases branch near Pyl-encoding MtmBs. **(A)** The evolutionary relations between 1,061 non-Pyl and Pyl encoding MtmBs were inferred with an approximate maximum-likelihood tree. Blue clades highlight methanogenic Pyl MtmBs (light blue) while bacterial and archaeal non-Pyl and Pyl MtmBs (dark blue) are contrasted to the clade housing the proposed tetramethylammonium methyltransferase (gold). The remaining enzymes were non-Pyl MtmBs of unknown function. **(B)** Expansion of a proposed tetramethylammonium/choline branch houses our MtqB sequence from *Methanococcoides methylutens* Q3c (red) highlights both bacterial and archaeal species largely as two branches, with multiple *Methanococcoides* and *Methanosarcina* sp. Purple dots were used to highlight reported strains for choline growth next to their protein accession number. Phylogenetic distance is measured by amino acid substitutions per site with scale bars for both **(A,B)**, and bootstrap values are represented as a gradient from 0% (blue) to 100% (gold).

We expanded out the sequence acquisition of all MtmB and MtxC sequences on NCBI to a minimal cutoff of 25% sequence identity (Figure 6, Supplementary Figure 2) to generate phylogeny for both protein families. We then explored records of QMA degradation to closest hits to ACT9XH_RS04775, then modeled those targets (Figure 5C) and explored the putative active site of that phylogenetic cluster (Figure 5D). Given the analysis of the MtqB homologs, it seems that there is complete conservation of both position 4 methionine and position 5 cysteine along with several aromatic residues near the QMA ligand. Regarding the phylogenetics of the MtxC family, each of the cognate corrinoid proteins appear for MttC (TMA), MtbC (DMA), and MtmC (MMA) but cluster independently, despite being in the same broad branch (Supplementary Figure 2). This is not the case for MtqC (QMA) which is annotated as a dimethylamine corrinoid protein and separate from the other classically annotated methylamine corrinoid binding proteins.

To finalize the molecular docking of the QMA/choline system, we sought to generate a co-structure of the MtqA and MtqC complex (Supplementary Figure 3). Upon docking the complex, we had a direct interaction between the thiol atom of CoM and cobalamin ligand, with subsequent methylation of CoM through MtqA leading to the penultimate step prior to methyl-CoM methylreductase reduction of methyl-CoM and production of methane.

### Biochemical validation of QMA demethylation by ACT9XH_RS04775

We examined the potential that ACT9XH_RS04775 is the previously described MtqB enzyme, by performing substrate dependent CoM methylation activity of Q3c cell-free extracts from cells grown on either QMA or TMA. Our proteomics data revealed that ACT9XH_RS04775 and ACT9XH_RS04770 were both highly differentially produced in QMA and choline cells compared to TMA grown cells (Figure 3). We therefore predicted that QMA extracts should show QMA activity, but TMA grown cell-free extracts should lack such a response. Our results confirmed that QMA:CoM methyl transfer activity was observed in QMA cell-free extracts but not in TMA grown extracts (Supplementary Table 1). Interestingly, QMA grown cell-free extracts, but not TMA grown extracts, displayed choline:CoM methyl transfer activity as well (Supplementary Table 1), suggesting ACT9XH_RS04775 could play a role in choline demethylation, supporting both proteomic and modeling data.

**Figure 7:**
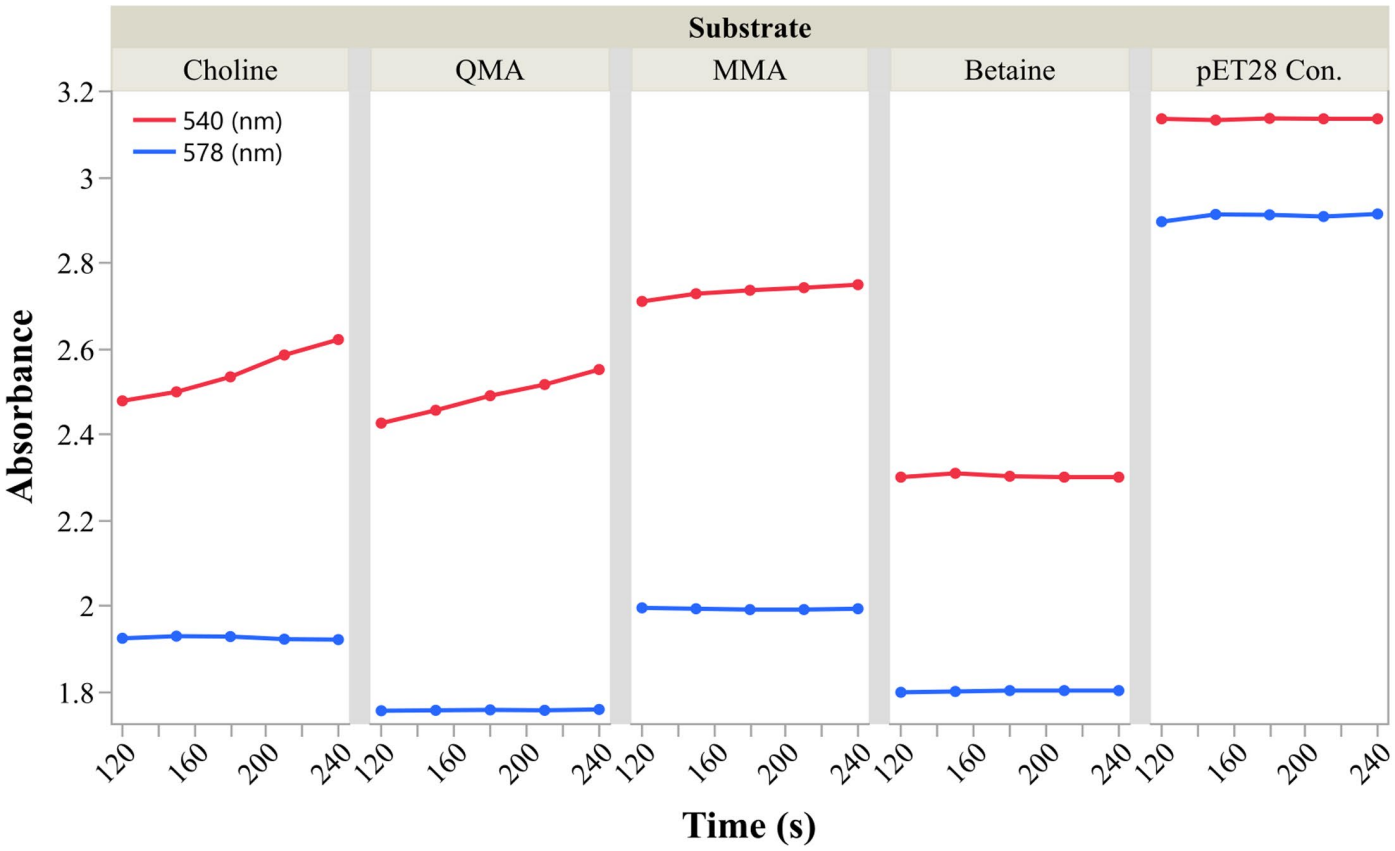
ACT9XH_RS04775 is a QMA and choline methyltransferase. Cob(I)alamin methylation activity of partially purified ACT9XH_RS04775 was tested *in vitro* using multiple substrates. The activity of the enzyme was monitored on each substrate for every 30 s for 120 s using an HP8453 photo diode array spectrophotometer. Increase in the absorbance at 540 nm (red) with no change in the absorbance at the 578 nm (blue) isosbestic point indicates substrate dependent direct conversion of free cob(I)alamin to methylcob(III)alamin. The pET28 control panel shows the results of the assay when supplemented with QMA but other substrates also showed no activity.

To further validate that ACT9XH_RS04775 is the MtqB, we cloned and expressed the gene in *Escherichia coli* BL21(DE3) and tested the activity of the partially purified enzyme. We were able to produce the hexahistidine tagged enzyme in *E. coli* BL21(DE3) and partially purify the enzyme by affinity chromatography with a Ni-NTA column. This enzyme preparation was able to catalyze the QMA-dependent methylation of free cob(I)alamin at a rate of 37 nmol min^−1^ mg^−1^, as evidenced by the time-dependent increase in absorbance at 540 nm while no increase in absorbance at the 578 nm isosbestic point was observed (Figure 7, Supplementary Figure 4). Based on the results from extracts above, we hypothesized that ACT9XH_RS04775 may also serve as the choline methyltransferase in Q3c. We therefore tested its activity and confirmed that it can also catalyze the demethylation of choline at a rate of 42 nmol min^−1^ mg^−1^. We tested the activity of the enzyme using MMA as the substrate, given that ACT9XH_RS04775 is a non-Pyl MtmB homolog, and surprisingly, a low level of activity of 11 nmol min^−1^ mg^−1^ was observed. No activity was seen with TMA and glycine betaine as substrates or in the minus substrate controls. Likewise, extracts of *E. coli* BL21(DE3) that had been transformed with empty pET28 vector and induced with IPTG showed no activity on any of the substrates, indicating that the activity was due to the presence of ACT9XH_RS04775 (Figure 7, Supplementary Figure 4). Our attempts to further purify ACT9XH_RS04775 resulted in instability and precipitation of the enzyme, which will be addressed in future studies.

The data suggest ACT9XH_RS07150 is the preferred methylcorrinoid:CoM methyltransferase (MtbA). ACT9XH_RS04775 is also conveniently located downstream of the Pyl operon, which would be required to catabolize the direct downstream intermediate TMA. ACT9XH_RS07150 production under TMA versus QMA is not statistically different (Figure 3). This is not surprising given that QMA demethylation leads to production of TMA, DMA, and MMA, which would all require an MtbA enzyme such as ACT9XH_RS07150. ACT9XH_RS10710 is the Pyl-containing MttB, which is the TMA:corrinoid methyltransferase and ACT9XH_RS10705 is its cognate TMA corrinoid-binding protein, under the conditions tested, and we do not see significant differences when compared with QMA given the next intermediate is TMA. In addition, ACT9XH_RS10715, annotated as a RamA corrinoid reductive activation protein, was produced under QMA-, choline-, and TMA-grown conditions (Figure 3D). RamA is required to reduce the corrinoid cofactor from the inactive Co(II) state to the active Co(I) state, enabling methyl transfer by MtqB/MtqC and other MtxB/MtxC systems (Ferguson et al., 2009; Huening et al., 2021). As a result, the organism produces these proteins, although they are more abundant in TMA conditions but not significantly (Figure 3D). These results support the results published by Asakawa et al. (1998), which showed the activity of the three component QMA-dependent enzymes, and have greatly contributed to the field by demonstrating that MtqB is the first annotated non-Pyl MtmB homolog categorized genomically, *in silico*, proteomically, and biochemically.

## Discussion

Our proteomic analysis of *M. methylutens* Q3c grown on QMA and choline versus TMA combined with biochemical analysis of the heterologously expressed MtqB have provided crucial insights into the enzymes involved in quaternary amine dependent methanogenesis. The results were particularly fascinating as they did not adhere to our initial hypothesis that non-pyrrolysine (non-Pyl) MttB homologs were responsible for all quaternary amine metabolism. The most striking observation is the high abundance of ACT9XH_RS04775 (MtqB) and ACT9XH_RS04770 (MtqC) in both QMA-grown and choline-grown cells compared to TMA-grown cells (Figures 3A,B). This differential expression suggests that these proteins are specifically regulated and involved in QMA and choline metabolism. Through this study, we concluded that the non-Pyl MtmB homolog ACT9XH_RS04775 and cognate corrinoid protein ACT9XH_RS04770 are analogs to and serve the same role as the previously described MtqB and MtqC from strain NaT1. This conclusion is based on both the high abundance of ACT9XH_RS04770 and ACT9XH_RS04775 in the proteome of QMA-grown cells, but not TMA-grown cells, their near-identical N-terminal sequence matches with the MtqB and MtqC from *Methanococcoides methylutens* NaT1 (Asakawa et al., 1998) (Figure 4) and the ability of ACT9XH_RS04775 to demethylate QMA *in vitro* (Figure 7, Supplementary Figure 4). Importantly, our study provides the first evaluation of these enzymes since the loss of strain NaT1 and greatly expands our understanding of the MtqB enzyme in terms of its identity as a non-Pyl MtmB homolog and its unusual promiscuity for multiple substrates. The finding that ACT9XH_RS04775 (MtqB) showed a higher specific activity for choline than QMA (Figure 7, Supplementary Figure 4) is intriguing, particularly in the light of the higher specific activity of choline:CoM methyl transfer than QMA:CoM methyl transfer in QMA grown cell extracts (Supplementary Table 1). This suggests that the preferred and perhaps original substrate for this enzyme may be choline rather than QMA. This also suggests that horizontal gene transfer of the *mtqBC* genes to a methylotrophic methanogen capable of growth on methylamines could allow that organism to acquire the ability to utilize both QMA and choline as new substrates. Future mechanistic and kinetic studies of this enzyme will help broaden our understanding of the dual nature of this enzyme.

With this new insight of non-Pyl MtmBs in QA demethylation and methanogenesis, the connections to Pyl-encoding pathways are quite intriguing. While the second methyltransferase, ACT9XH_RS07150 (MtqA/MtbA), shows similar abundance in both QMA and TMA-grown cells (Figure 3C). This is consistent with its proposed role as a methylcorrinoid:coenzyme M methyltransferase (MtbA), which would be required for processing all methylamine intermediates (TMA, DMA, MMA) produced during QMA demethylation, and once again, directly downstream of the Pyl operon essential to those intermediates. The high sequence identity (83.3%) of ACT9XH_RS07150 with the N-terminal sequence of MtqA from strain NaT1 further supports its role in this pathway. The Pyl-containing TMA-specific methyltransferase ACT9XH_RS10710 (MttB) and the corrinoid protein ACT9XH_RS10705 (MttC) show slightly higher abundance in TMA-grown cells, but the difference is not statistically significant (Figures 3E,F). This can be explained by the fact that QMA demethylation produces TMA, necessitating the production of TMA-specific enzymes even during growth on QMA. Our results not only confirm the enzymatic activities reported by Asakawa et al. (1998) but also provide a molecular basis for QMA metabolism in methanogens. The identification of MtqB as a non-Pyl MtmB homolog is particularly significant, offering new insights into the evolution and diversity of methylamine-utilizing pathways in these organisms.

ACT9XH_RS04775 (MtqB) being a non-Pyl MtmB homolog, contradicting our initial prediction, raises interesting questions on the environmental and evolutionary mechanism of these methyltransferases. This finding highlights a comparison between both choline and phosphocholine as there seems to be more at play regarding the R_4_ group moiety and gene family usage. Pyl-MtxB isomers distant from Pyl-encoding pathways have been shown to be related to nitrogen availability and regulation (Nayak and Metcalf, 2019), however, this has yet to be demonstrated with non-Pyl MtxBs as they do not typically yield direct nitrogen sources, but this may be invoked now through ACT9XH_RS04775 (MtqB) that leads to complete demethylation to ammonium for usage from QMA, but not choline. Non-Pyl MtmB enzymes have been known to exist for many years, but their functions and ecophysiological roles have remained elusive. Furthermore, the original hypothesis of *L-*pyrrolysine acting as a molecular mimic/positioning of substrates used in encoding enzymes is still retained through this discovery. What is most striking regarding MttBs are their stringent substrate specificity, however, MtqB is less so, as it performs QMA, choline, and a small amount of MMA demethylation.

We predicted the binding site residues in ACT9XH_RS04775 upon comparing the docking with QMA/B_12_ and choline/B_12_ versus the characterized Pyl-MtmB crystals from *Methanosarcina barkeri* (Hao et al., 2002) and observed that the binding centers were distinct (Figure 5B). Docking simulations with MtmB using QMA/B_12_ or choline/B_12_ did not result in binding to that catalytic site of MtmB, further suggesting its role as distinct to MMA which prior studies attribute to the necessity of Pyl. Furthermore, the close nature of the Pyl-ammonium adduct, and release of ammonium compared to the nitrogen of QMA and choline, suggests a very intriguing insight into substrate mimicry, previously suggested for GB and Pyl-TMA (Ticak et al., 2014). Given the size of the MtxB superfamilies, it is possible that pyrrolysine was selected for expansion of anaerobically underutilized carbon pools, e.g., simple methylamines to allow selective advantages toward methylotrophic methanogenesis, and more recently, within specific clades of anaerobic bacteria through tetrahydrofolate. It is also theoretically possible that through gene duplication and loss of pyrrolysine within the methyltransferases, selection pressure could drive diversification and usage of other quaternary amines that resembled close ligand positioning from the Pyl-adducts, which may be interesting to empirically evaluate through evolutionary studies. These docking predictions of interacting residues help pave the way for selecting the amino acids that can be mutated in the future to alter enzyme specificity and draw further insights into Pyl evolution.

This work further showed that the non-Pyl MtmBs are distributed across the superphylum of Asgard archaea, members of proteobacterium, and other strains of *M. methylutens*. Due to the stringent parameters used in this tree construction, there is a possibility of many candidates being filtered out as there could be other undiscovered non-Pyl MtmBs (Figure 6). We are establishing the foundation for exploring the biochemical mechanisms behind QMA and choline demethylation, environmental relevance, and the regulatory pathways associated with different methylamine substrates. Given the distance from Tokyo Bay to Virginia, it is interesting to ponder the significance of QMA ecologically and did this have more to do with choline? QMA, or its more common name tetramine, is often associated with shellfish and gastropods as both an osmoprotectant and toxin. More recently, some members of benthic foraminifera can perform total denitrification (Woehle et al., 2022), attributed to the symbiotic relationship of their microbial metagenome. Interestingly, some of these members encode MtqB-like homologs, further suggesting an interesting relationship between these genes, nitrogen utilization, and QMA ecologically. Furthermore, understanding the ecological and regulatory mechanisms governing enzyme expression in response to various methylamine substrates could reveal how *M. methylutens* Q3c adapts to different environmental niches. Our future work aims to explore the predicted substrate binding sites through selective mutagenesis and evaluating the methyltransferase activity and the relationship to Pyl. As ancestral genes undergo mutation and duplication, understanding these adaptations and selection pressures could uncover potential biotechnological applications, such as environmental remediation and biogas production from quaternary amine-containing waste streams.

## Data Availability

The datasets presented in this study can be found in online repositories. The names of the repository/repositories and accession number(s) can be found in the article/Supplementary material.

## Footnotes

1
https://rast.nmpdr.org

2
https://www.nite.go.jp/nbrc/dbrp/dataview?dataId=STNB0000000110458

3
https://massive.ucsd.edu/ProteoSAFe/dataset.jsp?accession=MSV000099751#:~:text=ftp%3A//massiveDftp.ucsd.edu/v11/MSV000099751/

4
https://www.genscript.com

5
https://www.ncbi.nlm.nih.gov/nuccore/NZ_CP199767.1
