# Supplementary material for: Methanogenesis from tetramethylammonium and choline in Methanococcoides methylutens Q3c requires a nonpyrrolysine monomethylamine methyltransferase homolog
Source: Front Microbiol. 2026 Mar 19;17:1739651. doi: 10.3389/fmicb.2026.1739651 (PMC13044021; doi:10.3389/fmicb.2026.1739651)
Supplement: Supplementary file 1 [file Data_Sheet_1.pdf]

Supplemental Figures:

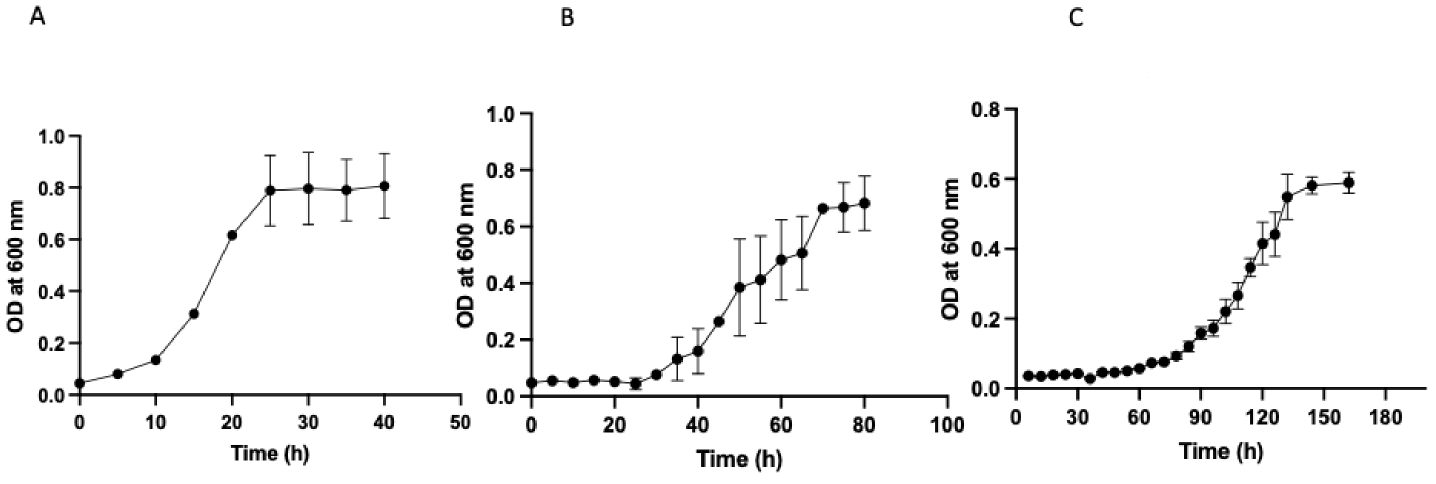

Supplemental Figure 1: Growth kinetics of *Methanococcoides methylutens* Q3c in brackish medium supplemented with trimethylamine, tetramethylammonium, or choline.
Growth curves (OD_{600}) are shown for Q3c cultures grown in brackish medium containing (A) 30 mM trimethylamine (TMA), (B) 30 mM tetramethylammonium (QMA), or (C) 30 mM choline. Data points represent the mean OD_{600} of replicate cultures, and error bars indicate standard deviation (SD). Cultures were harvested at mid-log phase for proteomic analysis at OD_{600} values of 0.60 (TMA), 0.40 (QMA), and 0.58 (choline). Maximum specific growth rates (µmax) were estimated from exponential-phase growth and are reported for each condition: TMA, 0.16 h^{-1}; QMA, 0.065 h^{-1}; choline, 0.036 h^{-1}.

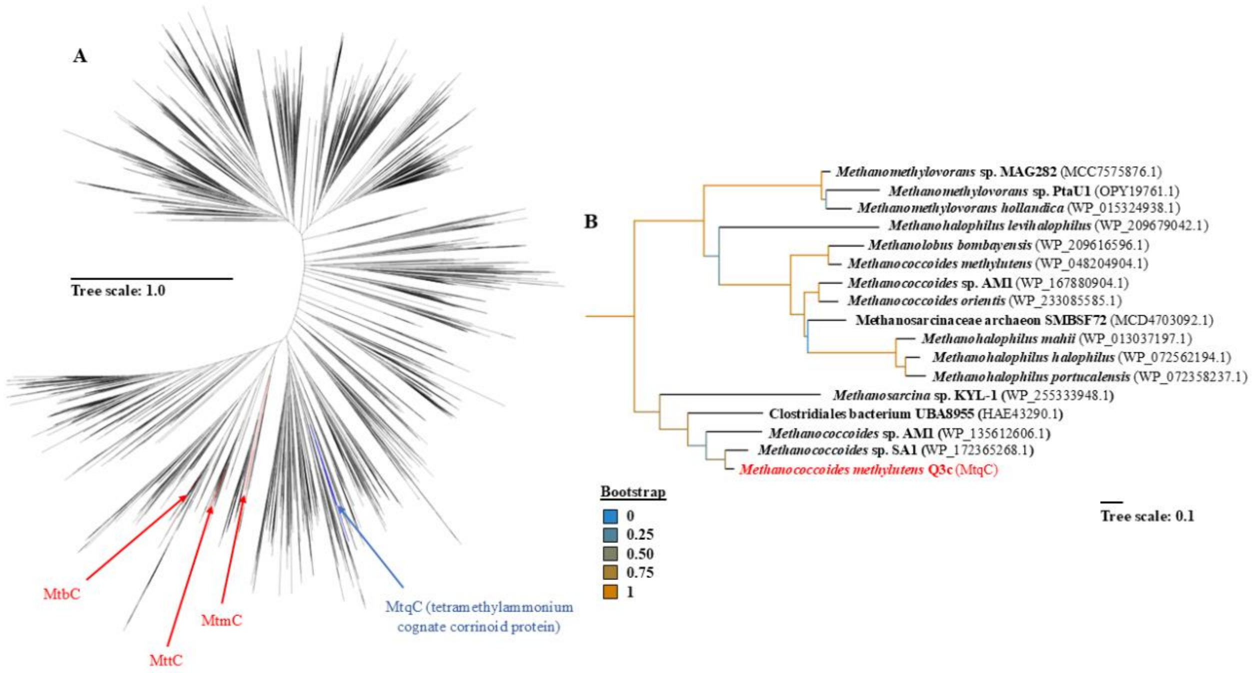

Supplemental Figure 2: Proposed tetramethylammonium corrinoid-binding protein phylogenetic relationship is distinct from MtxCs cognate to Pyl-encoding MtxBs. (**A**) The evolutionary relations between 2952 MtxCs were inferred with an approximate maximum-likelihood tree. Corrinoid proteins for monomethylamine (MtmB; *Methanosarcina acetivorans* C2A), dimethylamine (MtbC; *Methanosarcina acetivorans* C2A) and trimethylamine (MttC; *Methanosarcina barkeri* MS*)* are highlighted in red, in context to the positioning of tetramethylammonium corrinoid protein (MtqC; *Methanococcoides methylutens* Q3c) in blue. (**B**) The phylogenetic node harboring the proposed MtqC clade is expanded with ACT9XH_RS04770 (red) along with the names of encoding archaeal and bacterial sequences in bold text and accession numbers in parenthesis. Phylogenetic distance is measured by amino acid substitutions per site with scale bars for both (**A**) and (**B**), and bootstrap values are represented as a gradient from 0% (blue) to 100% (gold).

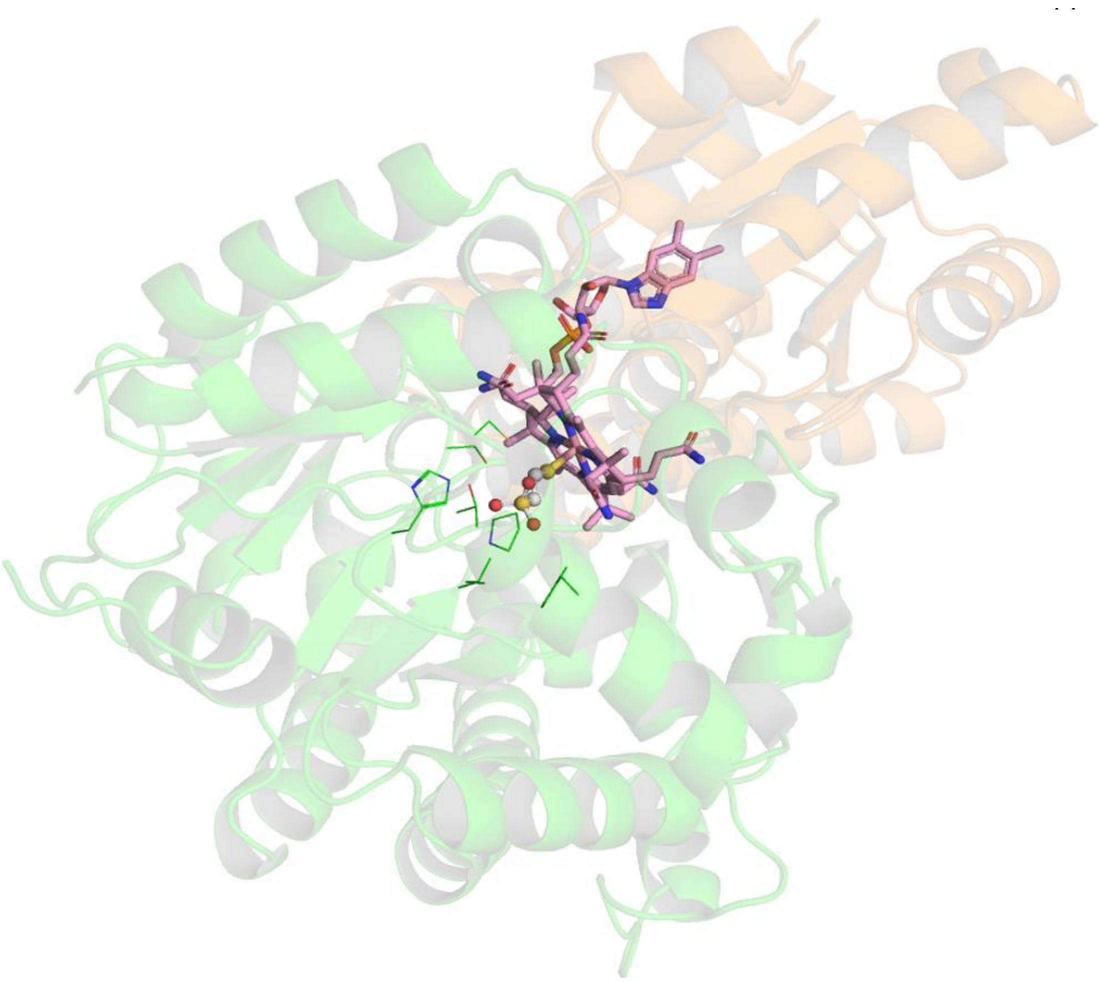

Supplemental Figure 3: MtqAC complex with coenzyme M and cobalamin. The amino acid residues for ACT9XH_RS07150 (MtqA/MtbA, green) and ACT9XH_RS04770 (MtqC, orange) were co-folded with both CoM (white ball and stick) and cobalamin (pink). Residues around 4 angstroms from CoM were shown as sticks.

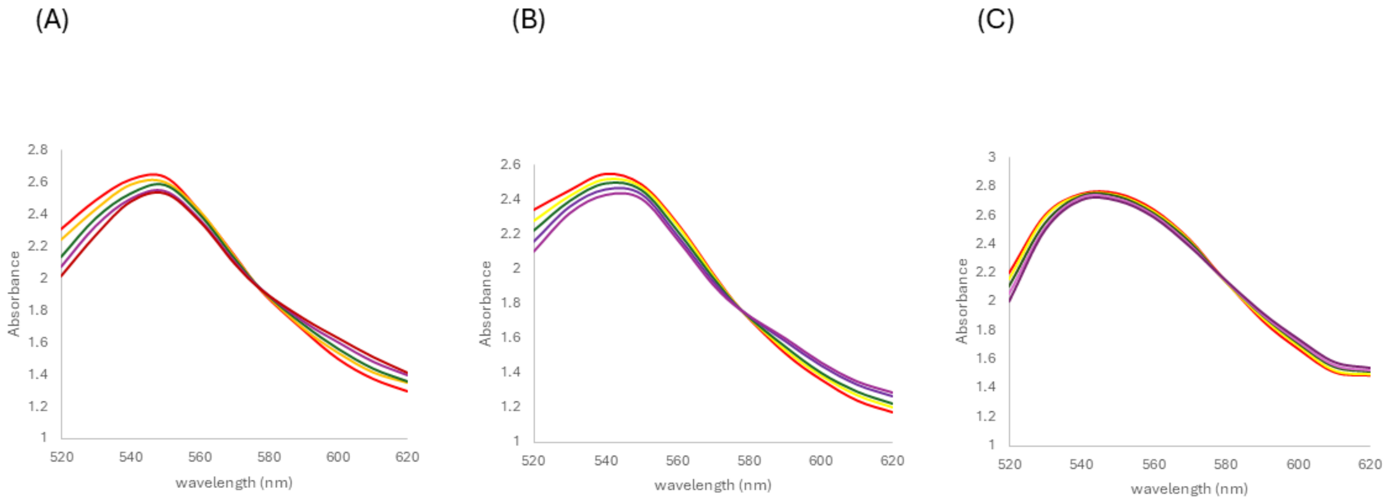

Supplemental Figure 4: Cob(I)alamin methylation activity of ACT9XH_RS04775 when provided with (A) choline, (B) QMA, or (C) MMA as a methylated substrate. Each spectrum represents a single time point n the assay taken every 30 sec using an HP 8453 photodiode array spectrophotometer. The absorbance increased over time at 540 nm and decreased over time at 620 nm while remaining constant at the isosbestic point of 578 nm, indicating direct conversion of cob(I)alamin to methylcob(III)alamin.

Supplemental Table 1: Substrate dependent coenzyme M methylation rates for cell-free extracts of *M. methylutens* Q3c grown on either tetramethylammonium or trimethylamine. Specific activities are reported in μmol CoM methylated min^{-1} mg^{-1} for the extracts when provided with the substrates listed.

	MMA	DMA	TMA	QMA	Choline
Extract of TMA grown cells	0.200	0.090	0.300	0.007	0.005
Extract of QMA grown cells	0.230	0.200	0.260	0.260	0.400
